# Selective Electroless Copper Plating of Ink-Jet Printed Textiles Using a Copper-Silver Nanoparticle Catalyst

**DOI:** 10.3390/polym14173467

**Published:** 2022-08-25

**Authors:** Golnaz Taghavi Pourian Azar, Sofya Danilova, Latha Krishnan, Yirij Fedutik, Andrew J. Cobley

**Affiliations:** 1 Functional Materials and Chemistry Research Group, Department of Engineering, Environment and Computing, Coventry University, Coventry CV1 5FB, UK; 2 Research and Innovation Services, University of South Wales, Pontypridd CF37 1DL, UK; 3 Plasmachem GmbH, 12489 Berlin, Germany

**Keywords:** electroless copper plating, selective metallisation, ink-jet printing, nanoparticle catalyst, e-textiles

## Abstract

The electroless copper plating of textiles, which have been previously printed with a catalyst, is a promising method to selectively metallise them to produce high-reliability e-textiles, sensors and wearable electronics with wide-ranging applications in high-value sectors such as healthcare, sport, and the military. In this study, polyester textiles were ink-jet printed using differing numbers of printing cycles and printing directions with a functionalised copper–silver nanoparticle catalyst, followed by electroless copper plating. The catalyst was characterised using Transmission Electron Microscopy (TEM) and Ultraviolet/Visible (UV/Vis) spectroscopy. The electroless copper coatings were characterised by copper mass gain, visual appearance and electrical resistance in addition to their morphology and the plating coverage of the fibres using Scanning Electron Microscopy (SEM). Stiffness, laundering durability and colour fastness of the textiles were also analysed using a stiffness tester and Launder Ometer, respectively. The results indicated that in order to provide a metallised pattern with the desired conductivity, stiffness and laundering durability for e-textiles, the printing design, printing direction and the number of printing cycles of the catalyst should be carefully optimised considering the textile’s structure. Achieving a highly conductive complete copper coating, together with an almost identical and sufficiently low stiffness on both sides of the textile can be considered as useful indicators to judge the suitability of the process.

## 1. Introduction

In the last few decades, interest in electronic textiles (e-textiles) and wearable electronics has significantly increased, which has stimulated development in these areas. Electronic components such as sensors, batteries or lights can be embedded into fabrics to add functionalities or decorative effects. This added value resulting from the integration of fabrics with electronics has been (and continues to be) beneficial to a wide range of applications, including, but not limited to, medical and healthcare, fashion, military, and workwear [1]. Textile-based memory devices [2,3], displays [4,5], solar cells [6,7] and energy storage devices [8,9] are all recent advancements in this field. To be able to interconnect multiple components of an e-textile, certain areas of the textile need to be electrically conductive. Generally, there are two main approaches to the creation of conductive textiles [10]. The first one is to integrate the conductive elements such as conductive filaments [1] or metal wires [11] into textiles by weaving, knitting, embroidering, etc. Alternatively, in this approach, the original yarn fibres can be covered by a metal [12] or conductive polymer [13] and then incorporated into the fabric. Although high conductivities can be achieved using these methods, the rigidity and inflexibility of the integrated conductive elements may have a negative impact on the wear comfort of the fabric [10]. Moreover, the produced e-textiles can easily snap or be damaged due to the different mechanical properties of those filaments and wires from the original fabric [11]. The second approach to make textiles conductive is coating of the textiles with a metal or conductive polymer after they are manufactured. Conductive coatings can be deposited using various techniques such as screen printing [14], ink-jet printing [15,16,17], sputtering [14] and electroless plating [18,19,20]. A common advantage of these techniques is that they can be used on finished textiles and are cheaper to process. Furthermore, due to the relatively thin layers of deposited coatings, the flexibility of the textiles is maintained. However, potential drawbacks of this approach are the adhesion of the coatings and the possibility for them to oxidise [21].

Among the methods to coat textiles with a conductive layer, electroless plating is a promising one due to its industrial feasibility, low cost, deposit uniformity and high conductivity [21]. In this technique, deposition of metal is achieved by immersing the substrate into a water-based solution. The main reagents in an electroless plating electrolyte are the salt of the metal, which is being deposited, a complexing agent which prevents spontaneous metal deposition and a reducing agent. The solution also contains a stabiliser and is formulated in a way that deposition should only occur on the activated substrate’s surface. Furthermore, a notable advantage of this process is its capability to metallise non-conductive materials, such as textiles. Electroless copper plating has been widely used in Printed Circuit Board (PCB) manufacturing to deposit a copper layer onto dielectric materials (such as epoxy-based polymers) [22].

In the case of non-conductive materials, for the electroless deposition reaction to initiate, the substrate needs to be activated [23]. The most commonly used activator is palladium, the role of which is to catalyse oxidation of the reducing agent. Once oxidised, the reducing agent donates electrons to the metal ion and the reduced metal deposits on the surface of the catalyst. Subsequently, the deposited metal acts as a catalyst and continues to catalyse the oxidation reaction, and therefore the deposit will continue to grow. Due to the increasing cost of palladium and its low abundance, researchers are now actively searching for alternative catalysts. For the electroless copper plating process, silver [24,25,26,27] and copper [20,28] nanoparticles are well studied as potential alternative catalysts. Both metals show catalytic activity towards the commonly used reducing agent—formaldehyde [29,30,31,32]. However, silver is still an expensive option, while copper is easily oxidised in air, hindering its catalytic activity. Previous research suggested that copper nanoparticles (Cu NPs) can be protected from oxidation by coating with silver metal. Moreover, some studies [33,34,35] showed that copper–silver composite nanoparticles have higher electrocatalytic activity compared to pure Cu or Ag NPs and could therefore be a promising catalyst for electroless plating. Synthesis of copper–silver core-shell nanoparticles (Cu_core_–Ag_shell_ NPs) can be performed in two steps: the first step is the synthesis of core Cu NPs, and the second step is the reduction of silver in the presence of Cu NPs. The reduction of silver from its salt is achieved by a displacement reaction where Cu^0^ is oxidised and donates an electron to Ag^+^, reducing it from its salt. As a result, the surface atoms of Cu NPs are replaced by silver [36,37,38].

To produce conductive tracks for e-textiles using electroless copper plating, the copper coating must be deposited on a certain area of the textile and not cover the whole surface. To achieve this, the catalyst should be deposited only on the required areas of the substrate. This selective deposition of the catalyst can be obtained using ink-jet printing [39,40], screen printing [41,42], microcontact printing [43,44] or using a gradient magnetic field [45]. Among the mentioned methods, ink-jet printing is a non-contact technique of catalyst deposition onto a substrate. The catalyst dispersion is the ink, which is stored in a cartridge. Once the process of printing begins, the ink is ejected from the printer head and drops onto the substrate surface under the force of gravity. The ink is deposited in a specific pattern, which is defined digitally by a CAD file. The main advantages of this approach can be summarized as (i) less contamination due to the lack of contact between the substrate and the equipment, (ii) lower consumption of ink, and (iii) the fact that the process does not require physical shape-transfer panels [46]. In the literature, there are studies focusing on the formulation of Cu_core_–Ag_shell_ NP-based inks for ink-jet printing [38,47,48]. In these inks, while Ag protects Cu from oxidation, using a Cu core significantly reduces the cost of the inks [49].

In our previous work [20], we investigated the efficacy of different functionalised Cu NP catalysts to initiate the electroless copper plating on polyester textiles. The conclusion from this work was that among different functionalising molecules, polyacrylic acid (PAA) was the most efficient one, which showed efficacy equivalent to or better than a palladium catalyst. In this study, in order to further increase the stability of Cu NP-PAA particles to oxidation, the catalyst was modified to contain Cu_core_–Ag_shell_ NPs. The developed catalyst was analysed using Ultraviolet/Visible (UV/Vis) spectroscopy and Transmission Electron Microscopy (TEM). The catalyst was then deposited selectively by ink-jet printing onto a polyester textile in different printing conditions. These variable conditions included the number of printing cycles (to change the catalyst NP loading) and the printing direction. The effect of the catalyst printing conditions on the subsequent electroless copper coatings was investigated regarding their visual appearance, copper mass gain, morphology, coverage and electrical resistance besides stiffness, laundering durability and the colour fastness of the respective metallised textiles. To realise this aim, Scanning Electron Microscopy (SEM), multimeter, stiffness tester and Launder Ometer were used.

## 2. Materials and Methods

### 2.1. Materials

The textile used in this study was Polyester Crepe de Chine White which was obtained from Whaleys Ltd. (Bradford, UK). This was a plain weave polyester with a thickness of 0.32 mm and mass/unit area of 120 g/m^2^. Ends per centimetre (EPC) and picks per centimetre (PPC) were 69 and 30, respectively. The chemicals for preparation of the Cu–Ag NP catalyst were purchased from Sigma-Aldrich (Taufkirchen, Germany), including copper (II) acetate monohydrate (98%), poly(acrylic acid, sodium salt) solution (average M_w_ ~8000, 45 wt.% in H_2_O), hydrazine (64–65%, reagent grade, 98%) and silver nitrate (ACS reagent, ≥99.0%). An ammonia solution (30% ROTIPURAN^®^), sodium hydroxide (≥98%, p.a., ISO, in pellets) and ethanol (≥96%, denatured) were obtained from Carl Roth (Karlsruhe, Germany). Proprietary chemistry including Circuposit Conditioner 3323A used to pre-treat the textiles and Circuposit 3350-1 used to prepare the electroless copper electrolyte were obtained from A-Gas Electronic Materials.

### 2.2. Equipment

For printing the catalyst, a small format thermal ink-jet BREVA printer with a printing stage of 15 cm × 15 cm was used. Standard empty HP45 cartridges were filled with the Cu–Ag NP catalyst and loaded into the printer. Ink cartridge settings were as follows: voltage of 13.3 V and pulse width of 2.7 µs.

### 2.3. Electroless Copper Plating Procedure Using Cu–Ag NP Catalyst

#### 2.3.1. Synthesis of Cu–Ag NP Catalyst

Cu–Ag NP colloids were synthesised in the following steps: (i) synthesis of Cu NPs; (ii) modification of the Cu NPs with polyacrylic acid (PAA); and (iii) formation of an Ag shell on the stabilised Cu NPs. The first two steps were carried out following the same procedure described in our previous work [20] to prepare the Cu NP-PAA colloid. Step (i) included using an aqueous solution of copper–ammonia complex and copper (II) hydroxide as the copper species in addition to the aqueous hydrazine solution. In step (ii), PAA was added to the Cu NPs to functionalise them, and an equal amount of ethanol was added to the reaction mixture afterwards. After stirring the mixture for 1 h, the particles were separated from the solution and washed by water and precipitated by ethanol. The Cu NPs were then re-dispersed in water using an ultrasonic bath for 1 h. The concentration ratio of the stabilised Cu NPs and PAA in the final colloid was estimated gravimetrically as Cu NP:polyacrylate~1:1.3–1:1.5.

To prepare the Ag shell in step (iii), a freshly prepared solution of 0.1 M AgNO_3_ in 2.5 M NH_4_OH was slowly injected drop by drop into the stirred Cu NP-PAA colloid. Following the addition of the first drop of the Ag solution, the Ag complex was directly reduced by the Cu atoms and Ag deposited on the surface of Cu NPs. This led to a colour change from the red wine (typical to Cu NPs) to brown red and deep black (characterised to Cu–Ag NPs). Cu–Ag NP colloids were initially synthesised in different Ag concentrations of 0.25–5 at.%, and following their analysis, the one with 5 at.% concentration was selected as the catalyst for this study.

#### 2.3.2. Printing of Cu–Ag NP Catalyst and Electroless Plating

**Figure 1:**
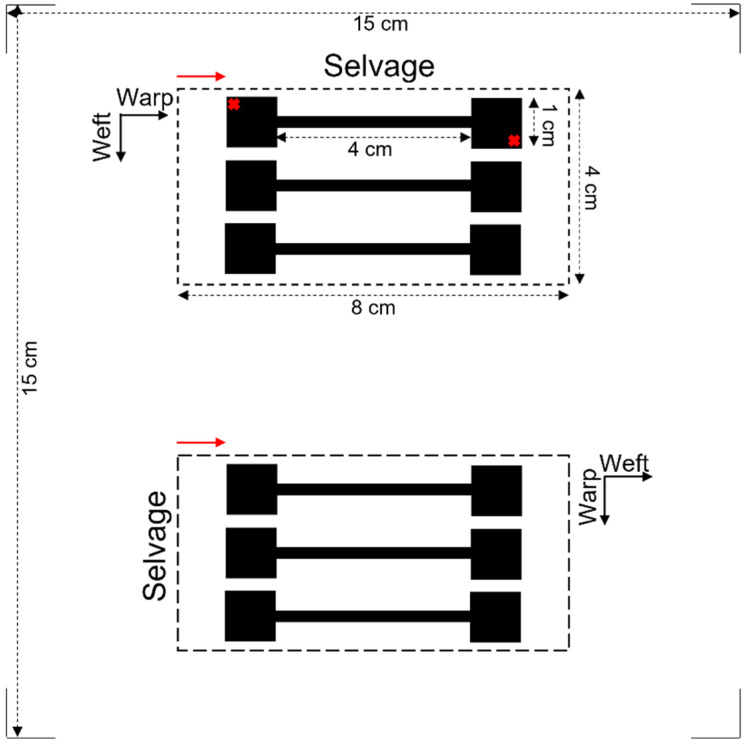
Designed printing image for characterisation of electroless copper coatings. Red arrows show the printing direction. Samples placed on the top and bottom were printed in the warp and weft directions, respectively.

The initial printing image designed for the characterisation of deposited electroless copper coatings is depicted in Figure 1. As it is observed, the total image size was 15 cm × 15 cm, and it included two identical rectangle patterns of 8 cm × 4 cm. This was selected in order to be able to centre the image (and therefore the samples) under the printer and simultaneously print two textile pieces in different directions. To achieve this aim, for each set of printing cycles, polyester textile was cut in two pieces of 8 cm × 4 cm in warp and weft way. In the warp way, one was cut such that the length of the sample was parallel to the selvage (so printed in the warp direction). In the weft way, one was cut so that the length of the sample was perpendicular to the selvage (so printed in the weft direction) (Figure 1). Textile samples were immersed in a Conditioner solution at 50 °C for 5 min. The solution was made up using Circuposit Conditioner 3323A according to the supplier’s guidelines. They were then rinsed under running water for 5 min followed by drying in the oven at 45 °C, typically overnight. Afterwards, samples were placed on the printer stage over the pre-marked 8 cm × 4 cm rectangles. Printing was completed using the as-synthesised Cu–Ag NP catalyst at different numbers of cycles (2, 3, 4 and 5) and each set was repeated 4 times. As a result, for each set of printing cycles, printing happened in the warp direction (for the sample placed on the top) and in the weft direction (for the sample placed on the bottom), simultaneously.

Subsequently, each pair of simultaneously printed samples were placed into an electroless copper electrolyte at 46 °C for 30 min. The electrolyte was made up using Circuposit 3350-1 according to the supplier’s guideline. The textile samples were then rinsed under running water for 5 min followed by drying in the oven overnight.

### 2.4. Characterisation of the Samples

#### 2.4.1. Characterisation of Cu–Ag NP Catalyst

In order to find the appropriate Ag concentration to protect Cu NPs, optical spectra were collected using a Hitachi U-2001 UV/Vis spectrometer from the diluted Cu–Ag NP colloids in different Ag concentrations (the optical length was 10 mm). To characterise the structure, size and distribution of the NPs in the Cu–Ag NP catalyst, a TEM technique was employed. For TEM analysis, a few drops of the dispersed Cu–Ag NP catalyst were pipetted onto a holey carbon film on a 200-mesh copper grid placed on top of a filter paper. This was followed by air-drying overnight, and then the grid was placed in the FEI Talos F200X TEM instrument (Thermo Scientific, Waltham, MA, USA) equipped with a super X EDS. The operating voltage was 200 kV and Velox software (Thermo Scientific, Waltham, MA, USA) was used to obtain the data.

#### 2.4.2. Characterisation of Electroless Copper Coatings

To measure the copper mass gain of the textiles, the mass of each piece of textile was measured once after cutting (in 8 cm × 4 cm) and then after electroless copper plating and drying in the oven overnight. The morphology of the copper coatings as well as the degree of the fibres’ coverage were characterised using a ZEISS GEMINI 500 VP SEM (Jena, Germany). To have a better insight into the conductivity of the metallised patterns from application point of view and in order to compare their performance in a more practical way, electrical resistance of the patterns was measured using a multimeter. The resistance was measured on the furthest corners of each dumbbell shape (red crosses in Figure 1), and the average resistance of all three shapes was reported as the resistance of each pattern.

#### 2.4.3. Textile Characterisations

Stiffness of metallised textiles was tested using a stiffness tester according to ASTM-D1388-96 standard [50] and measured using the cantilever principle. The sample size required for the stiffness tests was 15 × 2.5 cm. Therefore, the print image was designed by replacing the 8 cm × 4 cm rectangle areas in Figure 1 with fully black 15 cm × 2.5 cm horizontal strips. Two pre-treated textile samples were printed in the warp and weft directions simultaneously using the Cu–Ag NP catalyst at different numbers of printing cycles (2, 3, 4 and 5) followed by electroless copper plating for 30 min. Therefore, the sample printed in the warp direction was used to measure the warp way stiffness and the one printed in the weft direction was used to measure the weft way stiffness. A Shirley Stiffness Tester apparatus supplied from SDL Atlas Ltd. (Rock Hill, SC, USA) was used to measure the bending lengths of the metallised textiles. For each set of printing cycles, two readings on each side of the textile printed in the warp direction (face and back) and two readings on each side of the textile printed in the weft direction were taken as the warp way and weft way readings. The bending lengths and flexural rigidity of the samples were calculated using the Equations (1)–(3) [51]. Afterwards, the results were compared with the ones for an untreated control sample.

Bending length = L/2 cm (1)

where L—mean of the overhanging length of the specimen in cm

Flexural rigidity (G) = W × (L/2)^3^ mg·cm (2)

where W—weight per unit area of the fabric in mg/cm^2^

Overall flexural rigidity (G) = √G_warp_ × G_weft_ (3)

where G_warp_—Flexural rigidity in the warp way

G_weft_—Flexural rigidity in the weft way

The metallised textiles were washed according to the ISO 105-C06 (A1S):2010 standard [52] in a Launder Ometer from SDL Atlas Ltd. (Rock Hill, SC, USA). The washing tests were performed at 40 °C for 30 min using a standard detergent (ECE soap for ISO test) and material (textile) weight to liquor ratio of 1:50. For each set of printing cycles, three identical metallised samples printed in the warp direction, and three identical metallised samples printed in the weft direction were tested in up to five washing cycles. Samples were rinsed under running water after each washing cycle and then dried using a hot air dryer, and their resistance was measured using a multimeter. During the washing tests, the standard multifibre textiles were sewed to the samples as an adjacent textile, and the colour fastness was visually assessed using Grey Scales.

## 3. Results and Discussion

### 3.1. Characterisation of Cu–Ag NP Catalyst

During the synthesis of Cu–Ag NP colloids using the method applied in this study, Ag was deposited on Cu NPs as a result of a galvanic replacement happening spontaneously after Ag ions came into contact with the Cu NPs in the solution. Subsequently, while Cu atoms were oxidised into ions and dissolved in the solution, Ag ions were reduced into atoms and deposited on the surface of the Cu NPs. The sufficiently slow injection rate of the Ag precursor into the Cu NP colloid produced a low Ag ion concentration around the Cu NPs to ensure collision and nucleation of the Ag atoms on the Cu NPs. Therefore, our method of synthesis was expected to synthesise Cu_core_–Ag_shell_ NPs as the main product.

#### 3.1.1. UV/Vis Spectroscopy Characterization

**Figure 2:**
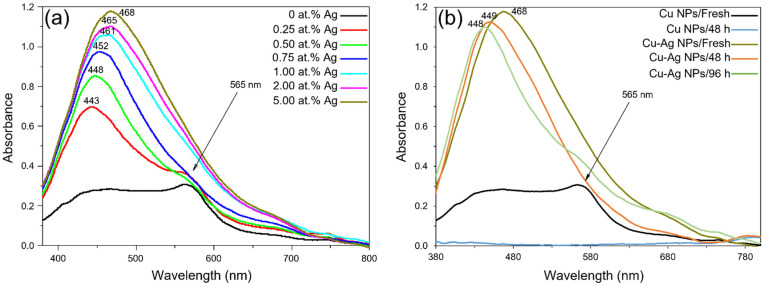
(**a**) Optical spectra of Cu–Ag NP colloids (diluted) at different silver concentrations; (**b**) optical spectra of Cu NP and Cu–Ag NP (~5 at.% Ag) colloids (diluted) at different times after their synthesis.

In order to determine the appropriate Ag concentration to protect Cu NPs against oxidation and to understand the architecture of the synthesised Cu–Ag NPs, UV/Vis spectroscopy was carried out on the diluted Cu–Ag NP colloids. Figure 2a shows the optical spectra of the synthesised Cu–Ag NP colloids at different Ag concentrations. The surface plasmon spectrum of the pure Cu NPs (black line) was characterised by the 565 nm copper plasmon peak only. After addition of Ag to the Cu NP colloid (red line), the absorption corresponding to silver appeared (at 443 nm). The observed absorption peak showed a red shift compared to the pure Ag NPs peak (at ~405 nm [53]). As the Ag concentration in the colloid increased, the absorption increased, and the absorption peak shifted more to the red region. According to Byeon et al. [53], the red shift and broadening of the surface plasmon resonance (SPR) band represent the presence of Cu–Ag bimetallic NPs as the dominant products. On the other hand, at higher concentrations of Ag, the 565 nm copper plasmon peak disappeared. UV/Vis spectra of binary metal or alloy NPs normally show two distinct surface plasmons. However, in the case of core-shell NPs, due to the formation of shell metal on the surface of the core metal, only one single peak at a wavelength close to the SPR peak of the shell metal might be observed. Therefore, it is likely that the synthesised Cu–Ag NP colloids with an adequate Ag concentration mostly contained Cu_core_-Ag_shell_ NPs. Similar results have been reported in other studies [36,37,53,54,55]. Furthermore, the presence of symmetric peaks and the sufficiently narrow surface plasmons indicated that most particles had a spherical shape [55].

One of the aims of this work was the formation of the Ag shell on Cu NPs to protect Cu NPs from air oxidation. Therefore, the oxidation behaviour of the synthesised Cu–Ag NPs was investigated for up to 96 h after their synthesis and compared with the behaviour of pure Cu NPs. Figure 2b shows the optical spectra of the synthesised pure Cu NPs and Cu–Ag NPs (with ~5 at.% Ag) over different time periods. It was observed that pure Cu NPs were oxidised and dissolved within 48 h after their synthesis (red line). However, Cu–Ag NPs were more stable since, after 96 h, the surface plasmon peak corresponding to the Ag shell was still present on the optical spectrum (light blue line). The observed blue shift of the silver plasmon peak with time and the appearance of a shoulder in the copper plasmon region (Figure 2b) could be related to the segregation of Cu and Ag in particles and oxidation of the Cu core [56]. Consequently, Cu–Ag NP colloid with ~5 at.% Ag was used for the later characterisations and then as a catalyst for the electroless copper plating of textiles.

#### 3.1.2. TEM Characterization

**Figure 3:**
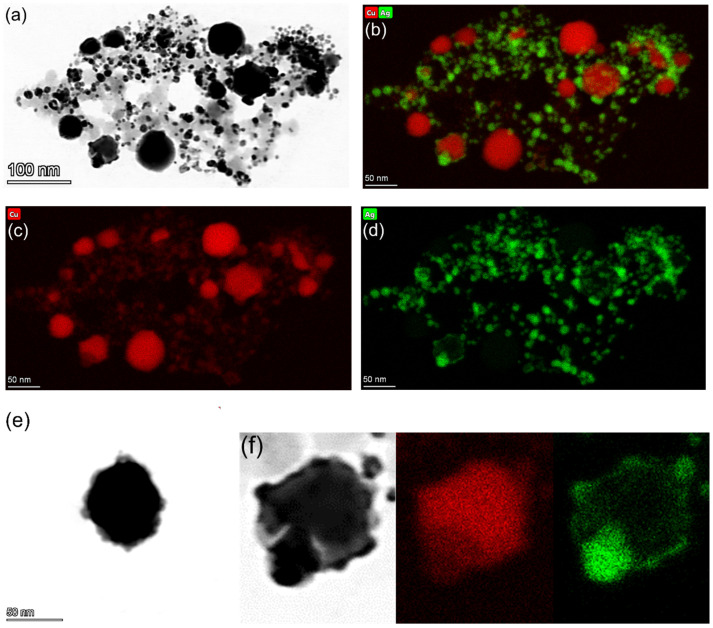
(**a**) TEM micrograph of the Cu–Ag NP catalyst; X-ray elemental mapping images of: (**b**) Cu & Ag; (**c**) Cu; and (**d**) Ag; (**e**) TEM image of a single Cu_core_–Ag_shell_ NP; and (**f**) partial sections of TEM image, and Cu and Ag X-ray elemental mapping images showing the higher contrast of Ag on the smaller particles and higher contrast of Cu on the larger particles.

The synthesised Cu–Ag NP catalyst was analysed using TEM to determine its structure in addition to the size and distribution of the Cu and Ag NPs (Figure 3). As it is observed in Figure 3a,b, in the Cu–Ag NP colloid, particles were present in different sizes including small NPs of 10–20 nm and larger NPs or particle aggregates of 60–70 nm. The mapping image of Cu distribution (Figure 3c) shows that large particles were clearly visible while smaller particles had weak contrast due to either lower mass and/or a partially shielded signal by Ag. However, the Ag distribution mapping image (Figure 3d) was more complicated. The size and shape of the Ag were generally similar to those of the Cu NPs showing high contrast on smaller particles whilst it was hardly detectable on large ones. This observation can be related to the higher surface to volume ratio of the smaller Cu NPs, which facilitates the collision of Ag ions and the nucleation of the Ag atoms to form the shell. This leads to a thinner Ag shell on large Cu NPs, which might not be easily detectable in TEM, although UV/Vis spectroscopy analysis confirmed its presence. Figure 3e shows a Cu NP with an approximate size of 70 nm having an Ag shell with an inhomogeneous thickness of ~1–7 nm. Thus, even at an equivalent thickness of the Ag shell, the Ag/Cu ratio drastically decreased with increasing Cu NP size. Figure 3f clearly shows the higher contrast of Ag on the smaller particles and higher contrast of Cu on the larger particles.

The location of the plasmon absorption bands of metal NPs depends on their size and shape, whereas for the core-shell NPs, it also depends on the thickness of the shell [57]. The observed Ag plasmon peaks lying in the range of 440–470 nm corresponded to pure Ag NPs with the size of 60–80 nm according to [58], which are much larger than the ones in Figure 3d. Therefore, the observed Ag plasmon peaks were probably related to the core-shell structure rather than being due to the presence of pure Ag NPs.

### 3.2. Characterisation of Electroless Copper Coatings

#### 3.2.1. Visual Inspection and Copper Mass Gain Measurements

**Figure 4:**
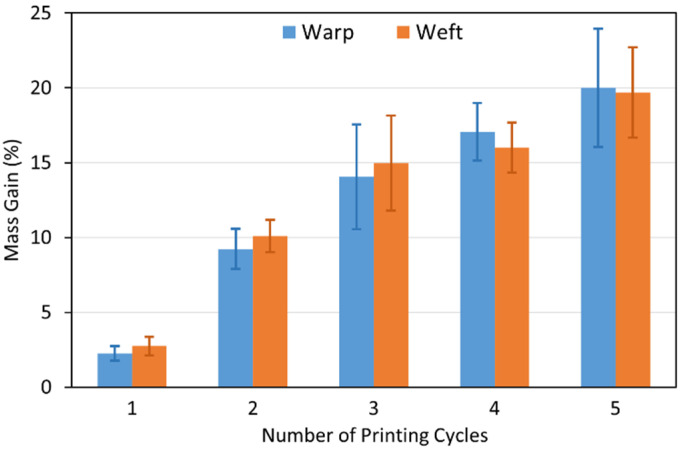
Electroless copper mass gain of textile samples printed with the Cu–Ag NP catalyst at different numbers of cycles and directions.

**Figure 5:**
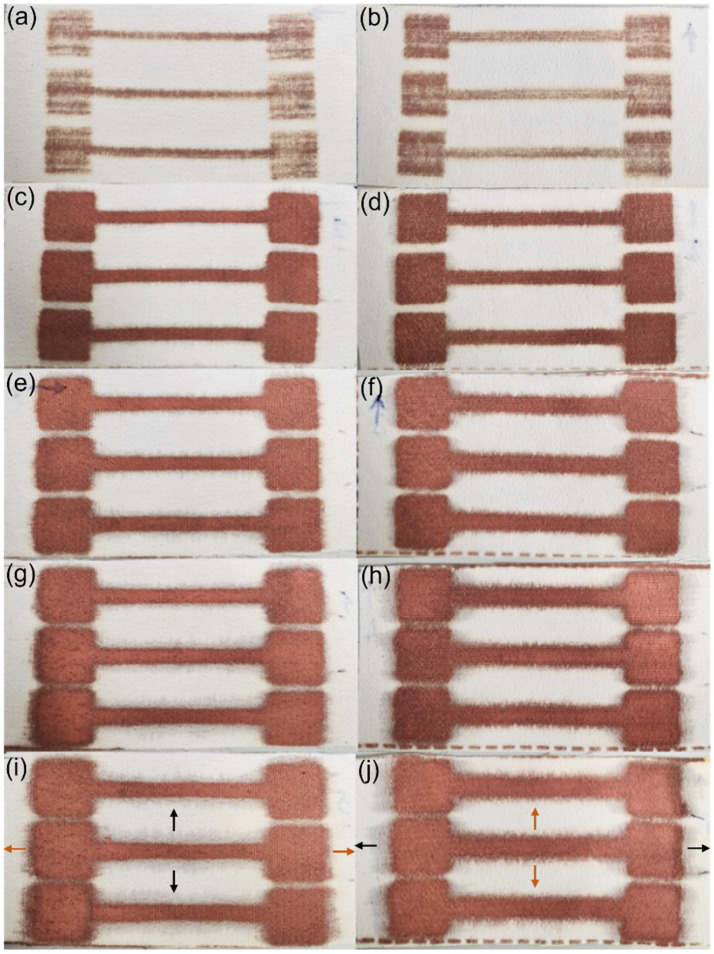
Photograph images of the metallised textiles printed with the Cu–Ag NP catalyst in the warp direction at: (**a**) 1; (**c**) 2; (**e**) 3; (**g**) 4; and (**i**) 5 cycles; and in the weft direction at (**b**) 1; (**d**) 2; (**f**) 3; (**h**) 4; and (**j**) 5 cycles.

Copper mass gain and appearance of the textiles after electroless plating are important indicators to compare the ability of catalysts (here different printing conditions of a catalyst) to facilitate electroless plating. In the case of our study, the aim of these analyses was to determine the effect of catalyst NP loading (different numbers of printing cycles) and printing direction on the deposited electroless copper coatings. Figure 4 shows the electroless copper mass gain of the samples, which were ink-jet printed with the Cu–Ag NP catalyst at different numbers of printing cycles and directions. As was expected, the copper mass gain increased as the number of catalyst printing cycles increased, although at higher printing cycles, the rate began to plateau. Each printing cycle introduced additional catalytic Cu and Ag NPs on the surface, which further facilitated the electroless copper plating, resulting in a higher copper mass gain. Figure 5 shows the appearance of the textiles (which had been ink-jet printed with the Cu–Ag NP catalyst at different numbers of cycles and directions) after electroless copper plating. The appearance of the samples was in agreement with the copper mass gain results verifying the higher amount of the electroless copper deposit over the patterns with increasing the number of printing cycles of the catalyst.

In ink-jet printing of a catalyst on textiles, metal NP loading is one of the most important parameters influencing different properties of the final metallised patterns. In the case of underloading, as the catalyst particles do not locate close enough to each other, a continuous electroless copper coating will not grow (Figure 5a,b). In addition, underloading of the catalyst may result in its shallower absorption by fibres. As a result, the subsequent electroless copper coating will only cover the surface rather than covering the fibres within the fabric. At the other extreme, overloading of a catalyst can lead to low adhesion of the deposited electroless copper coating on the one hand [45] and an excessive level of ink spreading (bleeding) on the other hand, resulting in poor pattern resolution.

Regarding the effect of printing direction, the copper mass gain of the samples printed with a catalyst at the same number of cycles in different directions was comparable after electroless plating (Figure 4). This result strongly suggests that printing direction of the catalyst has very little effect on the electroless copper mass gain. However, a very interesting finding of the visual inspection (Figure 5) concerned the spreading behaviour of the electroless copper coatings and its dependence on the printing direction. In both printing directions, as the number of printing cycles increased, spreading became more noticeable. On the other hand, at the same number of printing cycles, spreading was generally more significant on the textile printed in the weft direction. The metallised patterns printed with the catalyst in the warp direction had mainly sharper edges, and the dimensions of the patterns were closer to the original sizes. Similar results have been observed by Hajipour et al. [59] who investigated the effect of weave structure of the polyester fabric on the quality of ink-jet printing using a water-based ink. For the patterns printed in the warp direction, ink was diffused in the weft direction, and the density of the weft yarns floating over the warp yarns determined the ink spreading. On the other hand, when the patterns were printed in the weft direction, diffusion of the ink happened in the warp direction. They therefore concluded that it was the density of the warp yarns floating over the weft yarns, which controlled the ink spreading. Consequently, it is obvious that this type of ink spreading, which is perpendicular to the printing direction, occurs in both cases. However, in the case of the polyester textile used in this study, the density of the warp yarns was more than twice the density of the weft yarn (69 vs. 31/cm). Therefore, the ink spread more intensely perpendicular to the printing direction for the patterns printed in the weft direction. For the patterns printed in the warp direction, a grey shadow of ink spreading was observed in the weft direction (black arrows in Figure 5i) compared to the electroless copper plated perpendicular spreading (in the warp direction) on patterns printed in the weft direction (orange arrows in Figure 5j). The reason was probably the lower amount of catalytic ink spreading in the weft direction for the patterns printed in the warp direction, and this amount of the catalyst was not adequate to initiate the electroless copper plating over the spread area.

It is worth mentioning that not all the spreading happened perpendicular to the printing direction, and some ink spread in the printing direction. This type of spreading was more obvious on patterns printed in the warp direction as a result of the higher density of the warp yarns. This resulted in the copper plated spreading in the printing direction for the patterns printed in the warp direction (orange arrows in Figure 5i) compared to the grey spreading observed in the printing direction of the samples printed in the weft direction (black arrows in Figure 5j). Since our designed patterns were longitudinal, the area where the ink spread perpendicular to the printing direction was larger compared to the area where the ink spreading happened in the printing direction (36 cm vs. 6 cm). Therefore, in our case, it was the perpendicular spreading which controlled the total ink spreading resulting in a higher degree of the electroless copper coating’s spreading outwards of the patterns printed in the weft direction. In summary, ink spreading can be minimised by a careful selection of the textile’s structure and the printing conditions including printing design, printing direction and the number of printing cycles. This is important, particularly when printing circuitry to avoid short circuits. As can be seen in Figure 5c, the ink spreading was very low for the textile sample printed with the catalyst at two cycles in the warp direction. According to this figure, the width of the narrowest lines was almost identical to the original size on the printing image (2 mm). Therefore, lines as thin as 2 mm can be definitely printed and plated using this technique. Below 2 mm might also be possible although it was not tested in this study.

#### 3.2.2. Electrical Resistance Measurements

**Figure 6:**
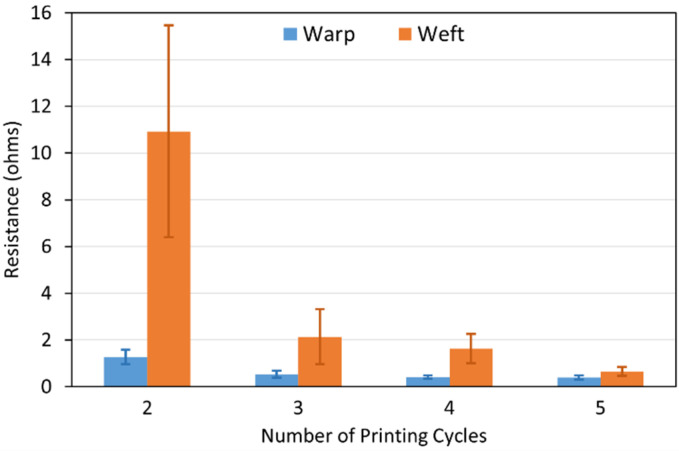
Electrical resistance of the metallised patterns printed with the Cu–Ag NP catalyst at different numbers of cycles and directions.

Electrical resistance of the electroless copper coatings on the polyester textiles printed with the Cu–Ag NP catalyst at different numbers of cycles and directions was measured using a multimeter. The results are depicted in Figure 6. The conductivity of the metallised patterns depends on the thickness, degree of coverage and depth of the electroless copper coatings. As can be observed in Figure 6, in both printing directions, resistance decreased as the number of printing cycles increased. This result was in agreement with the higher electroless copper mass gain for the increased number of printing cycles of the catalyst in both printing directions. On the other hand, at the same number of printing cycles, the resistance of the metallised pattern printed with the catalyst in the weft direction was higher than the one for the pattern printed in the warp direction. This happened even though both those samples had similar electroless copper mass gain (Figure 4). This observation was probably attributed to the higher overall degree of spreading for the metallised patterns printed with the catalyst in the weft direction (Figure 5) resulting in the lower chance of fibre coverage by electroless copper coatings (i.e., the same amount of deposit over a larger area). This result showed the important role of the catalytic ink spreading on the conductivity of the metallised patterns. According to Figure 6, the difference between the resistances of the electroless copper coatings printed with the catalyst at the same number of cycles and different directions decreased as the number of printing cycles increased. The metallised patterns which were printed with the catalyst at five cycles had almost identical resistance when printed in the warp and weft directions. At higher numbers of printing cycles, the catalytic ink had already saturated most of the fibres. Thus, spreading did not affect the fibres coverage by the electroless copper coating, and the subsequent conductivity was therefore similar.

#### 3.2.3. SEM Characterization

**Figure 7:**
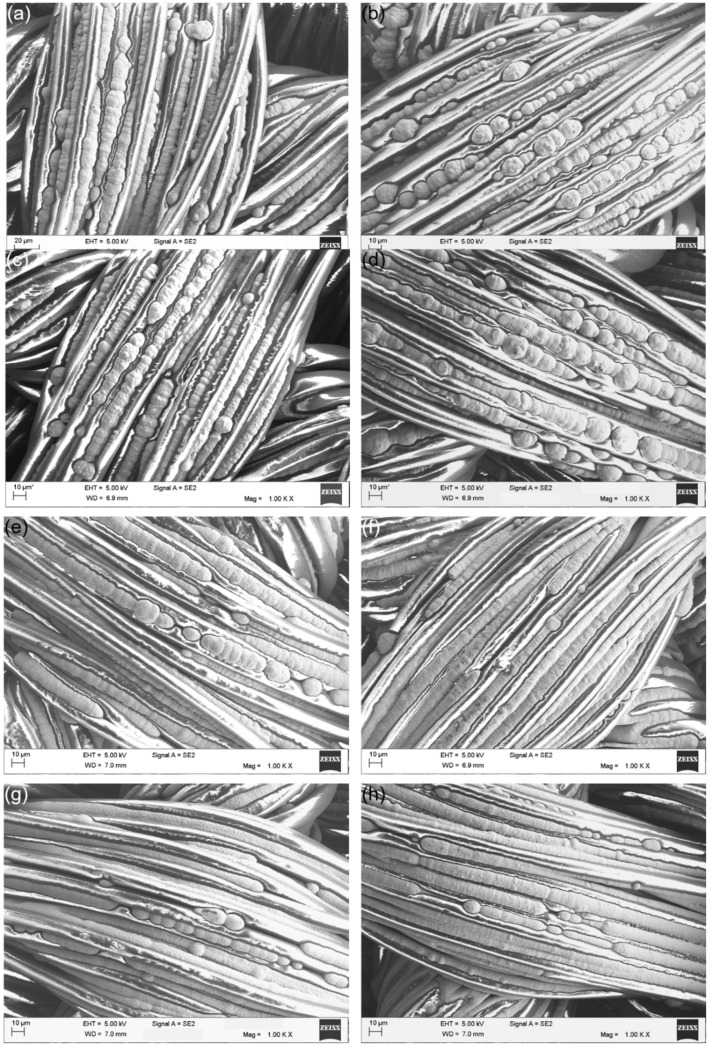
SEM micrographs of the metallised textiles printed with the Cu–Ag NP catalyst in the warp direction at (**a**) 2; (**c**) 3; (**e**) 4; and (**g**) 5 cycles; and in the weft direction at (**b**) 2; (**d**) 3; (**f**) 4; and (**h**) 5 cycles.

The metallised textiles printed with the Cu–Ag NP catalyst at different numbers of printing cycles and directions were characterised using SEM to study the coatings’ morphology and the level of the fibres’ coverage (Figure 7). Figure 7 shows that at the lower number of printing cycles, the electroless copper coatings had a nodular morphology (Figure 7a–d). However, with increasing the number of printing cycles, the level of nodularity of the coatings decreased, and they started to become smoother (Figure 7g,h). This was the result of an increased number of catalytic NPs on the surface at higher numbers of printing cycles leading to more nucleation sites. Consequently, electroless copper coatings were deposited which were more continuous and had a more uniform thickness. As it is observed in Figure 7, it was very difficult to realise any difference between the SEM images of the electroless copper coatings printed with the catalyst at the same number of cycles and different directions. Since the SEM micrographs were taken locally and at high magnification, it was nearly impossible to see any effect of coatings’ spreading and distinguish the resultant difference in the level of fibres coverage by the electroless copper coatings.

### 3.3. Textile Characterisations

**Figure 8:**
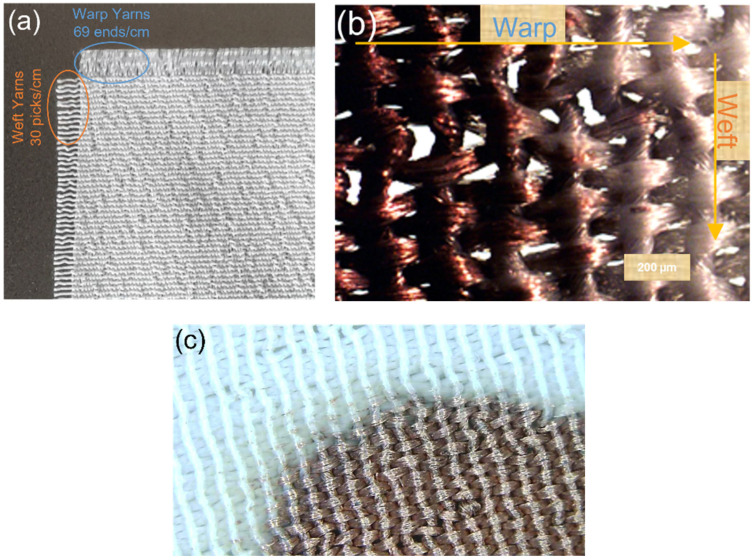
(**a**) Image of the untreated polyester textile under magnifying counting glass; (**b**) optical microscopy image of the metallised textile; and (**c**) optical microscopy image of the border between the metallised section and the non-metallised section.

As the first step to further characterise the structure of the textile being used in this paper, the untreated white polyester textile (the control sample) was examined under a magnifying counting glass (Figure 8a). The textile’s design was confirmed as a plain weave of 1/1 having finer warp yarns with 69 EPC and the coarser weft yarns with 30 PPC. Figure 8b shows the optical microscopy image of the metallised textile indicating the warp and weft yarns. Figure 8c depicts the optical microscopy image of the border between the metallised section and the non-metallised section, which gives some indication of the resolution which can be achieved for the electroless copper plating after selective catalysation of the textiles.

#### 3.3.1. Fabric Stiffness Test

**Figure 9:**
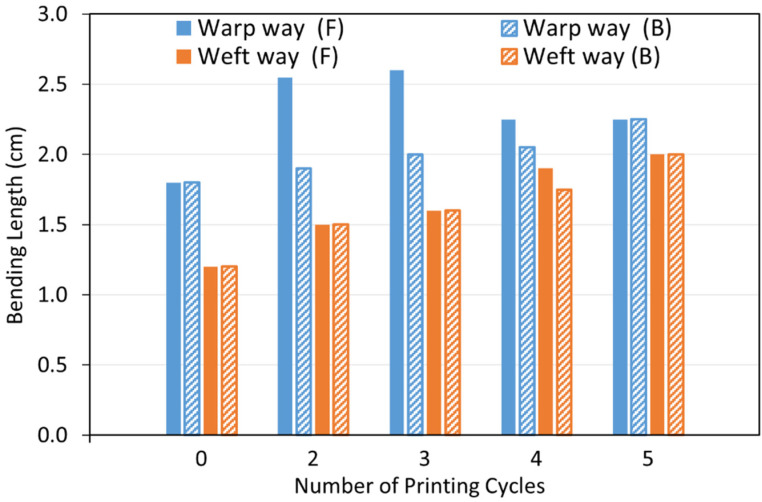
Bending lengths of the face (F) and back (B) side of the metallised textiles printed with the Cu–Ag NP catalyst at different numbers of cycles and directions.

**Figure 10:**
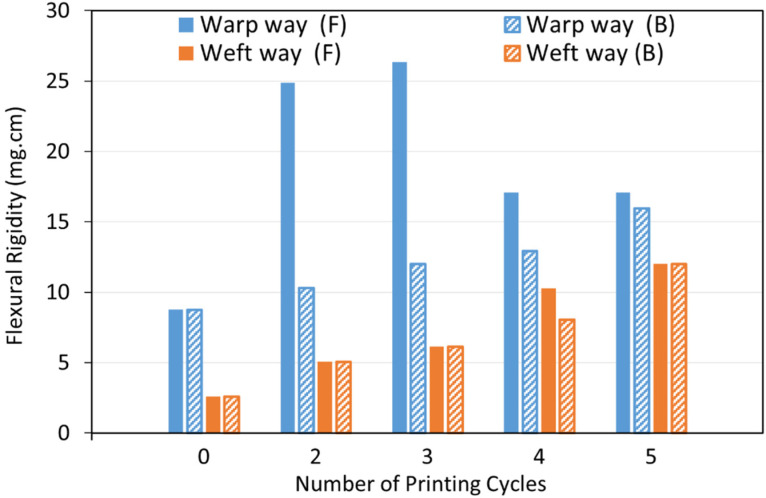
Flexural rigidity of the face (F) and back (B) side of the metallised textiles printed with the Cu–Ag NP catalyst at different numbers of cycles and directions.

**Figure 11:**
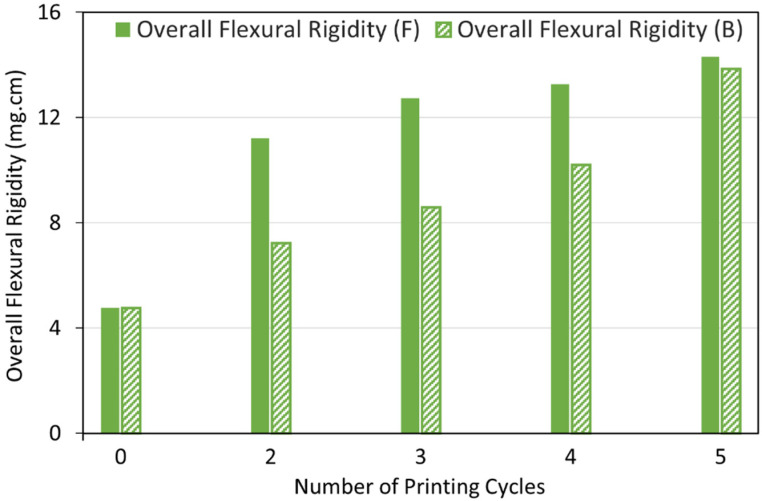
Overall flexural rigidity of the face (F) and back (B) side of the metallised textiles printed with the Cu–Ag NP catalyst at different numbers of cycles.

**Figure 12:**
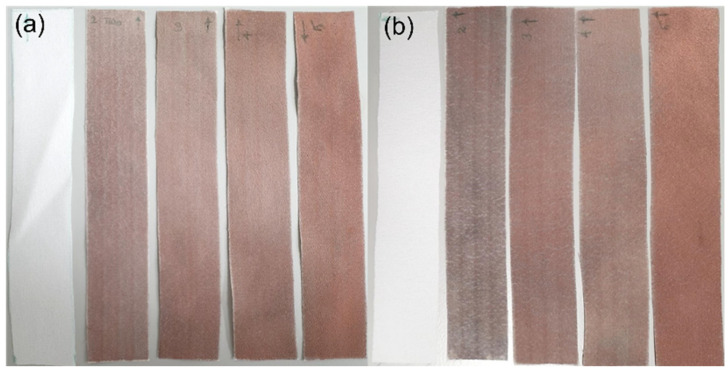
Photograph images of the back side of the metallised textiles printed with the Cu–Ag NP catalyst at different numbers of cycles (From left to right: untreated textile, 2, 3, 4 and 5 printing cycles) in the (**a**) warp; and (**b**) weft directions.

Selectively metallised textiles should be multifunctional and comfortable to wear to be able to serve as wearable electronics for different purposes. Stiffness is one of the most important characteristics of the textiles, which influences their flexibility, drape and handle properties. The bending resistance of the textiles is an indicator of their stiffness. To determine this, bending lengths of the metallised textiles printed with the Cu–Ag NP catalyst at different numbers of printing cycles and directions were measured using a stiffness tester. The results were compared with the ones for a control sample and are depicted in Figure 9. As it can be seen, the control sample had the same bending lengths on the face and back side of the fabric and a lower bending length for the weft way sample compared to the warp way one (Figure 9). As mentioned before, there was a difference in the warp way (finer yarns with 69 EPC) and the weft way (coarser yarns with 30 PPC) of the textile causing a difference in cover factor. Cover factor is one of the main parameters which affects the bending characteristics of the textiles. Stiffness of the textiles is mainly affected by the warp, weft, and overall fabric cover factor. In Figure 8a,b, the bigger gaps between the weft yarns compared to the smaller gaps between the warp yarns can clearly be seen, showing that the weft cover factor was less than the warp cover factor. This was the reason for the lower bending lengths on the face and back side of all the weft way metallised samples compared to the warp way ones, independent of the number of printing cycles of the catalyst. Figure 9 also shows that the bending lengths of the metallised samples printed with the catalyst in the warp direction (warp way bending lengths) were higher at 2–3 printing cycles with a big difference between the face and back sides. However, the bending lengths dropped at 4–5 printing cycles and became very similar on the face and back sides. It seems that at 2–3 printing cycles, most of the printed ink sat on the face side of the textiles (due to the higher cover factor) resulting in higher electroless copper deposit and higher bending lengths on the face side and a significant difference between the face and back sides. However, as they went through the higher printing cycles (4–5), the higher amount of the printed ink was enough for its deeper absorption from the face side to the back side of the textiles. This led to the higher saturation of the internal fibres by the catalytic ink followed by electroless copper plating on the back side of the textiles leading to similar bending lengths on both sides. Although polyester is hydrophobic in nature, the catalytic ink could penetrate to the back side of the fabric by capillary forces. Furthermore, for most of the number of printing cycles, there was no difference in the bending lengths on the face and back side of the metallised samples printed with catalyst in the weft direction (weft way bending lengths). This observation may be due to the lower weft cover factor leading to an easier migration of the ink from the face side to the back side of the textiles and similar level of electroless copper deposit on both sides. The values of flexural rigidity and the overall flexural rigidity of the metallised textiles printed with the catalyst at different numbers of cycles were calculated using Equations (2) and (3) in Section 2.4.3 and are plotted in Figure 10 and Figure 11, respectively. Figure 11 indicates that the overall flexural rigidity (stiffness) of the textiles was higher on the face side compared to the back side for each number of printing cycles and correspondingly increased with increasing the number of printing cycles on both face and back side. As was expected from the bending lengths results, the difference between the overall flexural rigidity of the face and back side of the metallised samples reduced at the higher number of printing cycles of the catalyst. For example, while the difference in the overall flexural rigidity of the face and back side of the metallised samples printed with catalyst at two cycles was 4 mg·cm, this difference was only 0.48 mg·cm for the metallised samples printed with catalyst at five cycles. This results in the electroless copper coating the printed face side of the textile and some of the internal fibres creating a coated conductive back side as well. For clarity, the images of the back side of the metallised textiles printed with the catalyst at different numbers of cycles and directions are shown in Figure 12. It can be observed that the increased number of printing cycles of the catalyst resulted in a more complete copper coating on the back side of the samples in both printing directions. The visible electroless copper coatings with high conductivities on the back side of the textiles together with an almost identical overall flexural rigidity of the face and back side of the metallised textiles (but low enough for comfort) are useful indicators to identify the successful printing conditions required to achieve conductive e-textiles with high performance. For the Cu–Ag NP catalyst used in this paper, from Figure 11, five printing cycles seemed to be the optimum to obtain the mentioned characteristics on the polyester textile used in this study.

#### 3.3.2. Laundering Durability and Fastness Test (Launder Ometer Test)

**Figure 13:**
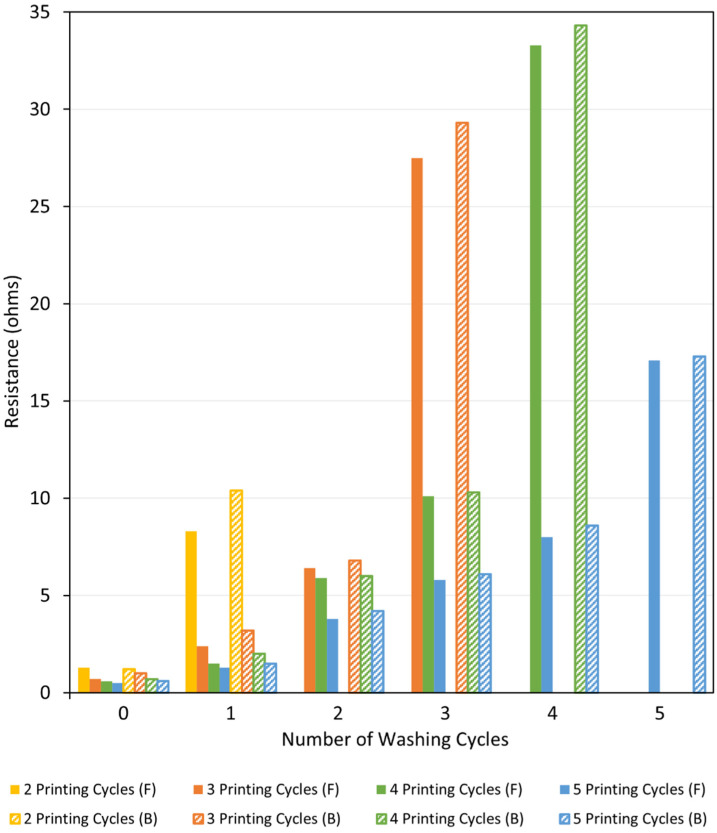
Resistance of the face (F) and back (B) side of the metallised samples printed with the Cu–Ag NP catalyst at different numbers of cycles in the warp direction versus number of washing cycles.

**Figure 14:**
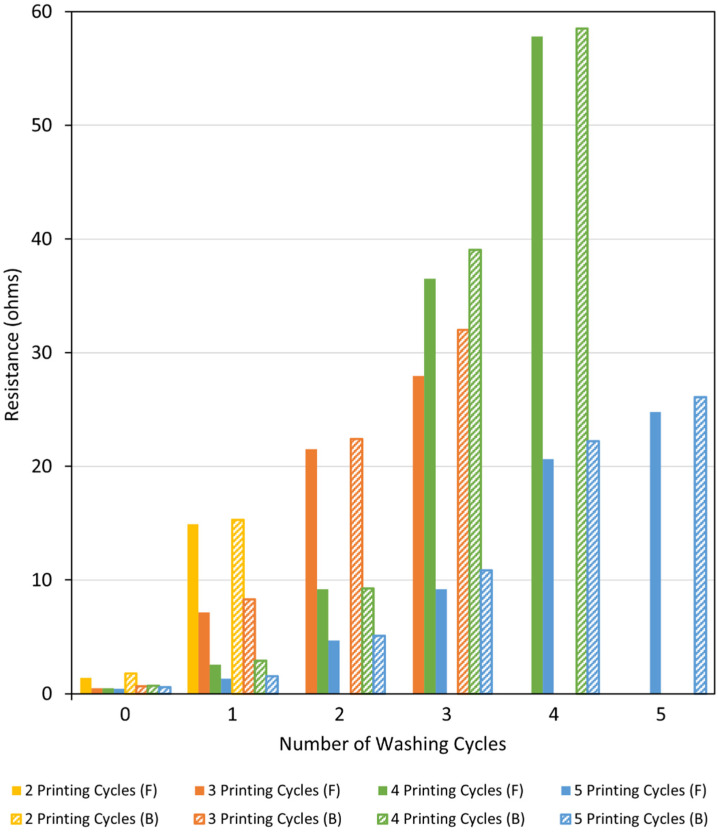
Resistance of the face (F) and back (B) side of the metallised samples printed with the Cu–Ag NP catalyst at different numbers of cycles in the weft direction versus number of washing cycles.

The metallised textiles printed with the Cu–Ag NP catalyst at different conditions were washed at different numbers of cycles. After each washing cycle, the electrical resistance on the face and back side of the metallised samples were measured using a multimeter. The aim was to observe the changes in their electrical resistance as well as the colour fastness to washing following each washing cycle. The results are shown in Figure 13 and Figure 14 for the metallised samples printed with the Cu–Ag NP catalyst in the warp and weft directions, respectively. Figure 13 indicates that the resistance values of the metallised patterns printed with the catalyst in the warp direction increased significantly with increasing the number of washing cycles, at different numbers of printing cycles. The same trend was also observed for the metallised samples printed with the catalyst in the weft direction (Figure 14). As Figure 13 shows, the resistance of the metallised textile printed with the catalyst at two cycles in the warp direction increased significantly at the face side of the fabric from 1.3 Ω to 8.3 Ω after one washing cycle. After that, the sample became non-conductive. The metallised sample printed at three cycles became non-conductive after three washing cycles, while the one printed at four cycles became non-conductive after four washing cycles. After the initial washing cycles, the resistance values increased moderately due to the removal of the unfixed copper particles from the textiles surface [60]. After that, the resistance values increased more significantly as a result of the repeated impact of the increased mechanical stress, water stress, temperature, and the influence of detergents [61]. As expected, in both printing directions, the resistance values were slightly higher on the back side of the metallised textiles when compared to their face side independent of the number of printing and washing cycles (Figure 13 and Figure 14). For the same number of printing and washing cycles, the metallised samples printed with the catalyst in the weft direction have shown higher resistance values compared to the ones printed in the warp direction. This was due to the initial higher resistance of the metallised samples printed with the catalyst in the weft direction compared to the ones printed in the warp direction for all number of printing cycles, before any washing test, as it was described in Section 3.2.2.

The laundering durability results confirmed the necessity of protecting the conductive tracks (the metallised surface of the textiles) in practical applications, against corrosion and wear damage resulting from the water stress, temperature, and mechanical stress of washing cycles. There are a variety of ways of protecting the electroless copper deposit including coating it with a polymer layer. Acrylate resin, polyurethane resin and polydimethylsiloxane have been proved to protect the copper plated tracks on the polyester textiles from wear resistance without affecting the track’s conductivity [62].

The ISO standard Grey Scales were used to visually assess the colour change on the washed samples and the colour staining on the white multifibre fabrics from the metallised samples after each washing cycle. The results are listed in Table 1. It was observed that independent of the number of cycles and direction of the catalyst printing or the number of washing cycles, there was no significant difference in the colour staining of all the metallised samples. The colour staining grade was 4/5, which was considered as Good. This indicated that there was only a slight staining on the white multifibre fabrics, which was acceptable. The source of this change in colour was probably due to the presence of metallic copper particles on the metallised samples. However, these particles did not adhere to the surface of the white multifibre fabrics. On the other hand, the lowest grade of the colour change (shade change) on the metallised samples at the increased number of washing cycles was three. This may be due to the influence of the detergent on the copper particles, water, and mechanical force during the washing cycles.

## 4. Conclusions

In this study, a functionalised the Cu–Ag NP catalyst was successfully synthesised and used as an ink-jet printing ink for the selective catalysation of the polyester textiles. The presence of a core-shell structure in the synthesised catalyst was proven to protect copper from oxidation. Different printing conditions were applied during ink-jet printing of the catalytic ink including varying the number of printing cycles and printing directions to see their effect on the final properties of the metallised textiles. Increasing the number of printing cycles enhanced the electroless copper mass gain in both printing directions. Although the textiles printed with the catalyst at the same number of printing cycles had similar electroless copper mass gain, the conductivities were lower on the ones printed in the weft direction, especially at lower numbers of printing cycles. This was probably due to the textile’s structure leading to higher total ink spreading on textiles printed with the catalyst in the weft direction. It was found that ink spreading can be minimised by careful selection of the textile’s structure and printing conditions including printing design, printing direction and number of printing cycles.

Due to the lower weft cover factor compared to the warp one, stiffness of the metallised samples was lower on the weft way at the same number of printing cycles. As a lower weft cover factor leads to the deeper absorption of the catalytic ink by fibres, the weft way stiffness was similar on the face and back side of the metallised samples regardless of the number of printing cycles. On the other hand, for the warp way metallised samples, stiffness was very different between the face and back side of the metallised samples at the lower number of printing cycles. However, it became similar at the higher printing cycles. Independent of the printing direction, metallised samples printed with the catalyst at five cycles had highly conductive, complete electroless copper coatings on their back side. For all numbers of printing cycles and directions, conductivity of the metallised samples decreased after each washing cycle. While the decrease was moderate for the initial washing cycles, it became more significant for the higher number of washing cycles. The colour staining grade was considered as Good (grade 4/5) for all the metallised samples while the lowest grade of the colour change only occurred at the increased number of washing cycles (i.e., three).

For the first time, the results of this study indicate the importance of understanding the textiles’ structure including the yarns thickness, density and the textiles cover factor in the warp and weft ways and how these influence the properties of the metallised textiles, such as conductivity, stiffness and laundering durability. Therefore, for each specific textile, printing conditions should be optimised to deposit the catalyst in such a way that can provide a metallised pattern with the desired properties to be suitable to serve as e-textiles.

## Figures and Tables

**Table 1:** Colour fastness of the metallised textiles printed with the Cu–Ag NP catalyst at different numbers of printing cycles and directions.

No. of Printing Cycles/Fastness	Washing Cycles									
	Warp Way Sample					Weft Way Sample				
	1	2	3	4	5	1	2	3	4	5
2/Colour change	4/5	4	3			4/5	3	3		
2/Colour staining	4/5	4/5	4/5			4/5	4/5	4/5		
3/Colour change	4/5	4/5	4			4/5	4	4	3/4	
3/Colour staining	4/5	4/5	4/5			4/5	4/5	4/5	4/5	4/5
4/Colour change	4/5	4	4	3/4		4/5	4	4	3/4	
4/Colour staining	4/5	4/5	4/5	4/5		4/5	4/5	4/5	4/5	
5/Colour change	4/5	4	4	4	4	4/5	4	4	3/4	3/4
5/Colour staining	4/5	4/5	4/5	4/5	4/5	4/5	4/5	4/5	4/5	4/5

## Data Availability

Not applicable.
